# Biphasic Temporal Relationship between Cancers and Systemic Sclerosis: A Clinical Series from Montpellier University Hospital and Review of the Literature

**DOI:** 10.3390/jcm9030853

**Published:** 2020-03-20

**Authors:** Léo Partouche, Radjiv Goulabchand, Alexandre Thibault Jacques Maria, Sophie Rivière, Christian Jorgensen, Valérie Rigau, Céline Bourgier, Didier Bessis, Alain Le Quellec, Isabelle Quere, Jacques Morel, Philippe Guilpain

**Affiliations:** 1 Montpellier’s School of Medicine, University of Montpellier, F-34967 Montpellier, France; l-partouche@chu-montpellier.fr (L.P.); radjiv.goulabchand@chu-nimes.fr (R.G.); a-maria@chu-montpellier.fr (A.T.J.M.); christian.jorgensen@inserm.fr (C.J.); v-rigau@chu-montpellier.fr (V.R.); Celine.Bourgier@icm.unicancer.fr (C.B.); d-bessis@chu-montpellier.fr (D.B.); a-lequellec@outlook.fr (A.L.Q.); i-quere@chu-montpellier.fr (I.Q.); 2 Department of Internal Medicine—Multi-organ Diseases, Local Referral Center for Auto-Immune Diseases, Saint-Eloi University Hospital, F-34295 Montpellier, France; s-riviere@chu-montpellier.fr; 3 University of Montpellier, IRMB, Inserm, CHU Montpellier (Saint-Eloi University Hospital), U1183 Montpellier, France; 4 Clinical Immunology and Osteoarticular Diseases, Therapeutic Unit, Lapeyronie University Hospital, F-34295 Montpellier, France; 5 Department of Biopathology, Biopathology Tumor Bank, Gui de Chauliac University Hospital, F-34295 Montpellier, France; 6 Department of Radiation Oncology, ICM-Val d’Aurelle, F-34298 Montpellier, France; 7 INSERM, U1194, IRCM, F-34298 Montpellier, France; 8 Department of Dermatology, Saint-Eloi University Hospital, F-34295 Montpellier, France; 9 Department of Vascular Medicine, Saint-Eloi University Hospital, F-34295 Montpellier, France; 10 EA 2992 Dynamic Cardiovascular Inconsistencies, Montpellier University, Nîmes, 34095 Cedex 2, France; 11 Department of Rheumatology, CHU and University of Montpellier, 34090 Montpellier, France; j-morel@chu-montpellier.fr

**Keywords:** Systemic sclerosis, scleroderma, breast cancer, lung cancer, cancer occurrence

## Abstract

Cancer among patients with systemic sclerosis (SSc) would appear to be more prevalent than in the general population. Pathophysiological hypotheses are multiple, involving intertwined factors such as immune system antitumoral response, oxygen species dysregulation, and immunosuppressive treatments. We aimed to identify SSc patients with cancer monitored at our center, describing their clinical and immunological characteristics, such as cancer-specific outcomes. We focused in particular on the temporal relationships between cancer onset and SSc diagnosis. A retrospective study was conducted on SSc patients from Montpellier University Hospital from 2003 to 2018. Clinical characteristics and outcomes of each SSc patient with cancer were recorded. Fifty-five patients with SSc and at least one cancer was included (median age 56 years (47–66)), with a median follow-up time of 11 years (4–15). Sixty-four metachronous malignancies were identified (12 patients had two cancers). Among them, early-onset cancer occurrences (±5 years from SSc diagnosis) included 23 cancers (39% breast cancers, 13% lung cancers, and 13% gastro-intestinal tract cancers). Twenty-two cancers occurred 10 years (±5 years) after SSc diagnosis (14% breast cancers, 23% gastrointestinal (GI) tract cancers, and 18% lung cancers). Patients without any of the two autoantibodies (anti-centromere (ACA) and anti-topoisomerase (ATA-scl70) antibodies) were more prevalent in the early-onset cancer subgroup (14 vs. 6, *p* = 0.02). This study brought to light two peaks of cancer occurrence in SSc patients. Early-onset cancers were associated with SSc with a specific immunological signature. Late-onset cancers might be the consequence of a subtle interplay between repeated target organ inflammation, immunosuppressant use, mesenchymal cell dysfunction and subsequent genetic alterations.

## 1. Introduction

Systemic sclerosis (SSc) is a rare autoimmune disease with severe organ involvement (notably heart, lung, and gastro-intestinal involvement), a poor vital prognosis and severe long-term disability. The three major pathophysiological mechanisms are immunity dysregulation, uncontrolled fibrogenesis and vascular impairment. Additionally, oxidative stress dysregulation is also involved. These mechanisms are also implicated in the pathophysiology of cancer, and recent data suggest that cancer and SSc may share common determinants (genetic, epigenetic alterations) [1]. Firstly, an overall higher risk of cancer incidence (mainly breast and lung cancers) is described among SSc patients [2]. Secondly, tumor antigens may trigger autoimmunity in a subgroup of patients with specific autoantibody signatures (such as circulating anti-RNA Polymerase 3 (RNA-PolIII) or anti-RNA binding Protein Containing 3 (RNPC) antibodies) [3,4,5]. In these patients, genetic alterations (somatic mutations and/or loss of heterozygosis) are present within the gene coding for the target antigen expressed in the tumor, leading to a cross-reactive immune response.

These results prompted us to describe the demographical, clinical, and immunological features of SSc patients from the Montpellier area who also presented cancer in their medical history (before, after or simultaneously), with a particular interest in the chronological sequence of these events.

## 2. Material and Methods

Study design and population: we collected patients diagnosed with systemic sclerosis and cancer from 2003 to 2018. Systemic sclerosis and cancers were identified through the disease-coding system at Montpellier University Hospital. Patients from the departments of internal medicine, vascular-medicine, and rheumatology were identified and included through local medical databases.

Inclusion criteria: adult patients were included when fulfilling American College of Rheumatology/European League Against Rheumatism (ACR/EULAR) SSc diagnosis criteria [6]. The disease subtype (limited SSc and diffuse SSc) was categorized according to LeRoy et al. and assessed by experts in SSc [7]. Sine scleroderma SSc patients were included, whereas patients with localized scleroderma, morphea, and Buschke scleroderma were excluded.

Data recording: all medical charts were double reviewed by experienced internists, for the comprehensiveness of the data collection and in order to check the consistency of the data. Clinical, laboratory, and radiological features were collected from Montpellier hospital medical software (DxCare®, v 7.7.5, Clamart, France), and from the medical charts of Montpellier Cancer Institute.

Studied criteria: for each SSc patient, we recorded the following items: sex, age at SSc onset (first symptom, for example Raynaud’s phenomenon); disease subtype; organ involvement: skin ulcerations, interstitial lung fibrosis, pulmonary arterial hypertension (PAH), gastroesophageal reflux disease (GERD), chronic intestinal pseudo-obstruction (CIPO), renal crisis, cardiomyopathy; autoantibody status: antinuclear antibody, anti-topoisomerase 1 antibody (ATA-scl70), anticentromere antibody (ACA) (RNA-polymerase-III antibody measurement was not available for all patients during the study period); treatments; cancer characteristics; date of latest follow-up; date of death. General clinical data were also recorded when available: past medical history; tobacco use; alcohol use; associated autoimmune diseases (systemic lupus erythematosus, anti-phospholipid syndrome, rheumatoid arthritis, Sjögren’s syndrome, autoimmune hepatitis, primary biliary cirrhosis). Different characteristics of cancer were recorded: anatomical site; age at cancer diagnosis; histological features; cancer stage; cancer treatment strategy (surgical, chemotherapy, radiotherapy, hormonotherapy, or immunotherapy); cancer prognosis. We studied each occurrence of cancer independently. Hence, metachronous cancer events within a single patient were considered as two cancer occurrences; conversely, synchronous cancers at the same anatomical site in a single patient were considered and analyzed as one cancer occurrence.

We compared general characteristics of SSc patients with cancer to a cohort of consecutive SSc patients with exhaustive clinical, demographical and immunological data.

Statistical analysis: continuous data are presented as median (interquartile range (IQR) (25th percentile, 75th percentile) or mean. Categorical variables were expressed as absolute numbers and percentages. Statistical analysis was performed using the chi-squared test or Fisher’s exact test (when the number of events was under 5 in one of the subgroups) for categorical variables. *p*-values of less than 0.05 were considered significant.

Ethical approval: this project has been validated by our local International Review Board (2019_IRB-MTP_12-25).

## 3. Results

### 3.1. General Characteristics of Systemic Sclerosis (SSc) Patients with Cancer

We identified 55 patients with systemic sclerosis and at least one cancer, among a cohort of 358 patients followed in our center. Characteristics of patients, diseases and treatments are presented in Table 1 and Table 2. There were 32 patients with limited SSc (58.1%), 20 with diffuse SSc (36.4%), and 3 with SSc sine scleroderma (5.5%). Twenty-five of these (45.5%) had at least one severe organ involvement (interstitial lung fibrosis, pulmonary arterial hypertension (PAH), renal crisis, or cardiomyopathy), leading to the use of an immunosuppressive treatment for 22 of them (40%). Nineteen of these patients were tobacco users (19/31, 61.3%).

Eighteen patients had received immunosuppressive therapy for SSc prior to cancer onset (48.6%): methotrexate was the most prescribed within this subgroup (13 patients).

Immunosuppressive drugs had been given before the diagnosis of cancer in 2 (28.6%) of breast cancers, 3 (60.0%) of blood malignancies, and 4 (100.0%) of urinary tract cancers. Details are given in Supplemental Table S2b.

Compared to a group of 152 consecutive patients with complete data records, the characteristics of these 55 SSc patients with cancer were different in univariate analysis for: (i) the median age of patient at SSc diagnosis (46 years old within the group without cancer, versus 56 years old in the group of patient with cancer, *p* = 0.0002); (ii) the clinical subtype of SSc (more limited SSc in the group without cancer, *p* = 0.0037); (iii) and the death rate (6 deaths versus 19 in the group with cancer, *p* < 0.0001).

### 3.2. Characteristics of Cancer

Among these 55 patients, 67 cancers were identified. 12 patients had two cancers in their medical history. For 9 of them, the two cancers were metachronous. 3 of them had synchronous cancers at the same anatomical site (Supplemental Table S1b): (i) a 68-year-old patient with limited SSc had had bilateral synchronous breast cancers 21 years before diagnosis of SSc (Patient 1); (ii) one had bilateral synchronous lung cancers 26 years after limited a SSc diagnosis (Patient 12); (iii) one 47-year-old man with diffuse SSc had synchronous oesophagus cancer and cholangiocarcinoma 10 years after diagnosis of SSc (Patient 10).

**Figure 1:**
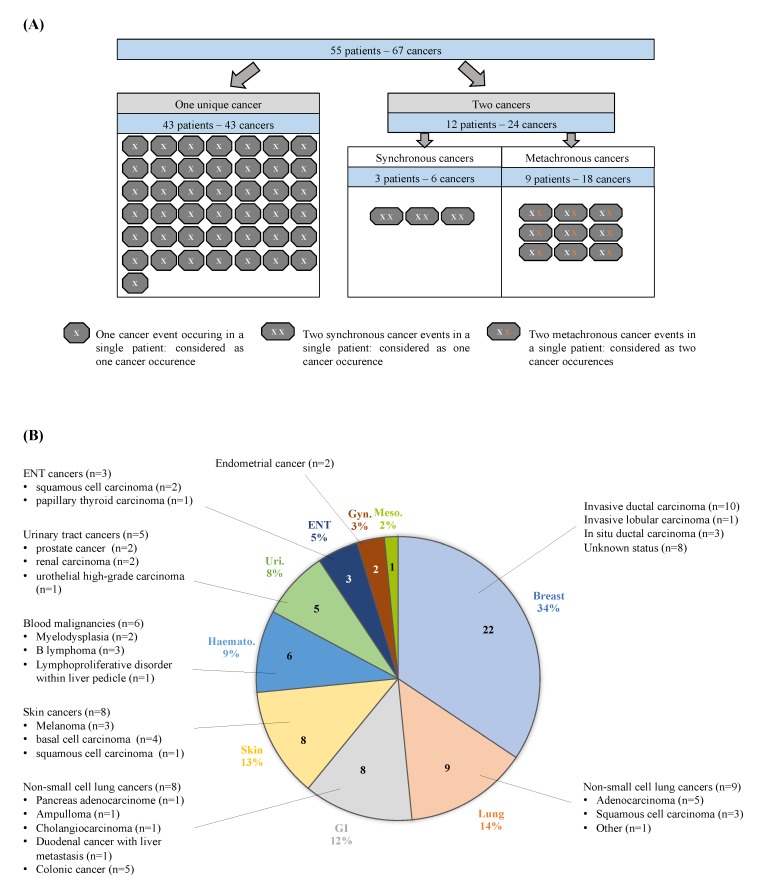
General characteristics of cancer events. (**A**) Distribution of cancer events within the SSc population: metachronous cancer events within a single patient were considered as two occurrences of cancer (white cross symbolizes the first cancer, while orange cross symbolizes the second cancer); synchronous cancers in a single patient were considered as one occurrence of cancer; 64 cancer occurrences were analyzed within 55 SSc patients. (**B**) The distribution of cancer occurrence subtypes (*n* = 64) and cancer histological subtypes within SSc patients (*n* = 55); GI: gastro intestinal tract cancers; Haemato.: haematological cancers; Uri.: urinary tract cancer; ENT: ear, nose and throat cancers; Gyn.: gynaecological cancers; Meso.: mesothelioma.

Consequently, we considered 43 cancer occurrences among 43 patients with one cancer, 3 cancer occurrences among 3 patients with synchronous cancers within the same anatomical site, and 18 cancer occurrences among 9 patients with 2 metachronous cancers. We thus studied 64 occurrences of cancers among these 55 patients (Figure 1A). Cancer subtype distribution and histological subtypes are shown in Figure 1B.

### 3.3. Temporal Relationship between SSc and Cancer Diagnoses: Two Peaks of Occurrence

**Figure 2:**
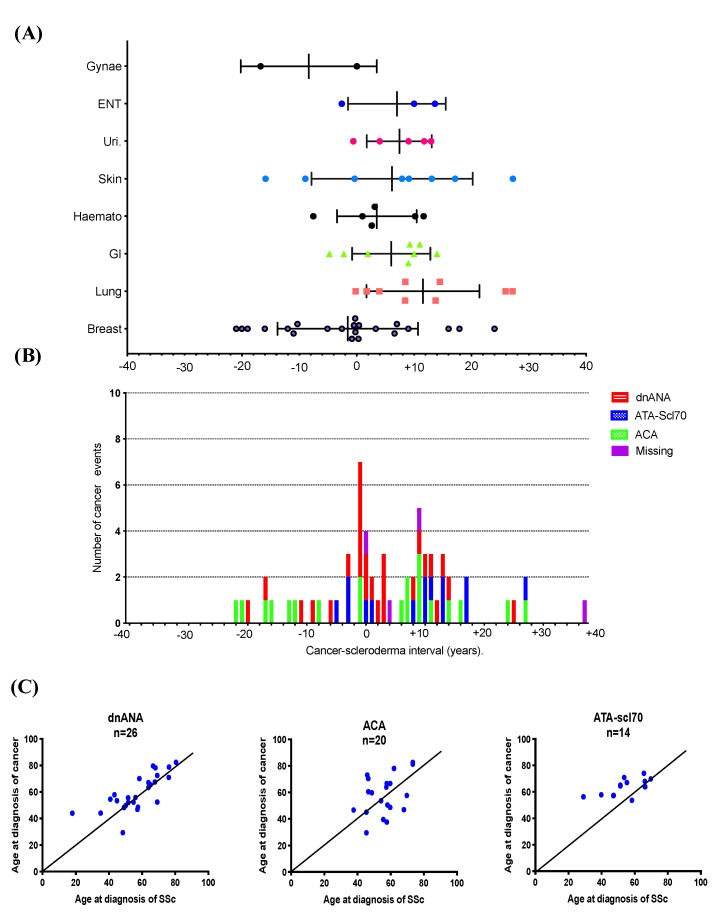
Temporal relationship between systemic sclerosis and cancer. (**A**) Temporal relationship between cancer onset and SSc diagnosis according to cancer subtypes (T_0_: time when SSc was diagnosed; ENT: ear, nose and throat cancers; GI: Gastrointestinal tract cancers; Haemato: Haematological cancers; Uri: urinary tract cancers; Gynae: gynaecological cancers). (**B**) Cancer-SSc time according to SSc antibody subtypes. (**C**) Age at cancer occurrence and age at SSc diagnosis according to autoantibody subgroup. Pearson’s correlation coefficient according to antibody subtype: dnANA R^2^ = 0.5496; ACA R^2^ = 0.1593; ATA-scl70 R^2^ = 0.3819; SSc: systemic sclerosis; dnANA: double-negative antinuclear antibody; ACA: anticentrome antibody; ATA-scl70: anti-topoisomerase 1 antibody.

We thus considered 64 cancer occurrences separately. The delay between SSc onset and cancer diagnosis according to each cancer subtype is shown in Figure 2A. Hence, breast cancers seem to be diagnosed before or close to SSc diagnosis whereas lung cancers and GI tract cancers are diagnosed later.

Over the graphical timeline (Figure 2B), two peaks of cancer occurrence are highlighted: the first one takes place around SSc diagnosis (±5 years), and the second one takes place 10 years after (±5 years). As shown in Figure 2B, this first peak of cancer occurrence contains 14 cancer events for which SSc was associated neither with the ACA nor ATA-scl70 antibodies (dnANA). 

Moreover, 26 occurrences of cancer concerned patients with antinuclear antibodies (ANA) positivity without ACA or ATA-scl70 antibodies (dnANA) (46.2%). In this subgroup of “dnANA” patients, the cancer event period and the SSc diagnosis period were very close as shown in Figure 2C.

#### 3.3.1. First Peak: Close Temporal Relationship between SSc and Cancer Diagnosis (±5 years)

**Figure 3:**
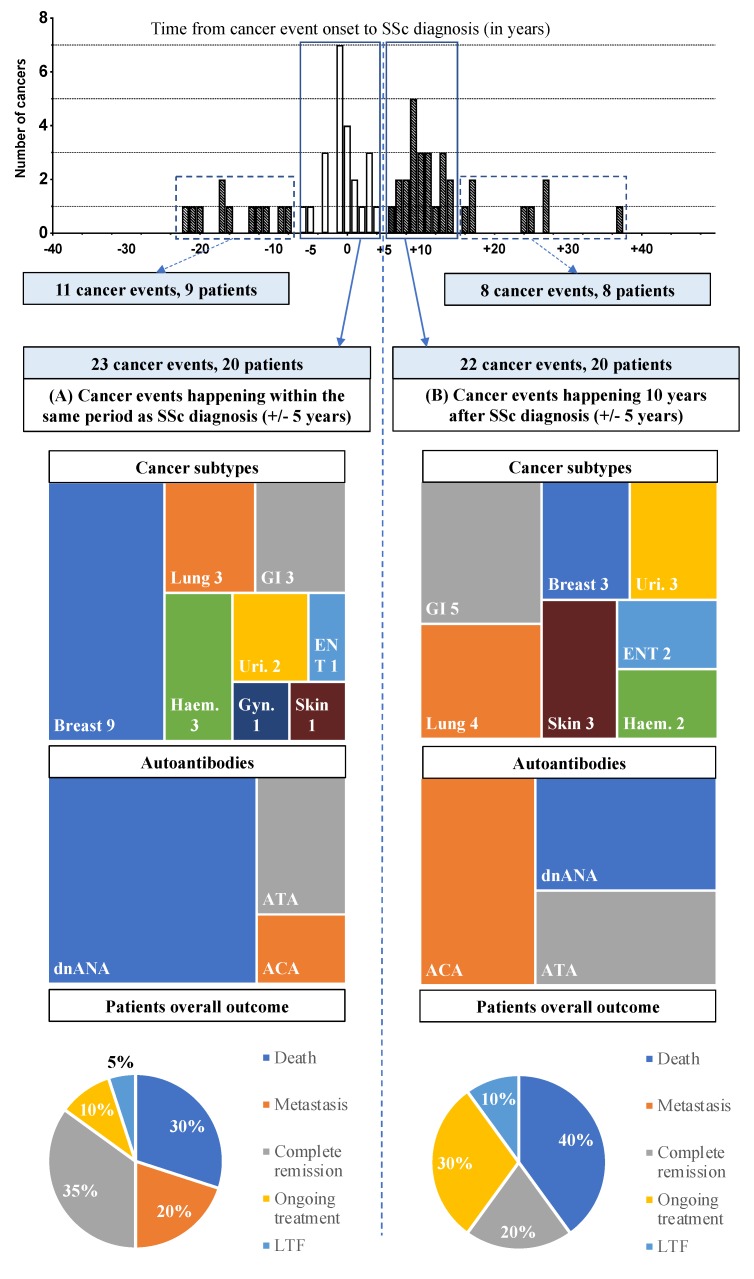
Description of cancer subtypes, autoantibody profiles, and global outcomes: Comparison of early-onset cancers (**A**) and late-onset cancers (**B**); the characteristics of cancer diagnoses widely spaced from SSc are described in the results section. SSc: systemic sclerosis; GI: gastro intestinal tract cancers; Haem.: haematological cancers; Uri.: urinary tract cancer; ENT: ear, nose and throat cancers; Gyn.: gynaecological cancers; ANA: Antinuclear antibody; ACA: anticentromere antibody; ATA: anti-topoisomerase 1 antibody (anti-scl70); dnANA: double negative ANA; LTF: lost to follow-up.

The first peak of cancer occurrence within our population concerned 23 cancers (20 patients), occurring within the same period as SSc diagnosis (±5 years). Among them, 9 were breast cancers, 3 were lung cancers, 3 were gastro-intestinal (GI) tract cancers (2 colon cancers, 1 liver cancer), 2 were urinary tract cancers (1 kidney and 1 prostate cancer), 1 was a nasopharyngeal cancer, 3 were blood malignancies (2 myelodysplasia, chronic lymphoid leukemia), 1 was an endometrial cancer, and 1 was a melanoma (Table 2, Figure 3).

The main characteristics of these cancers occurring in SSc patients are shown in Table 2, and cancer event characteristics and outcomes are shown in Figure 3. Within this subgroup, at the end of the follow-up 20% of patients (4 patients) had metastases, 3 of whom died from cancer; four patients are in complete remission, 1 patient is undergoing follow-up with hormonotherapy.

Breast cancers were the most frequent cancer within this first peak. They concerned 9 women, with a median age at SSc diagnosis of 65 years (52–68) and a median age at cancer diagnosis of 64 years (52–68). Six of these patients had a temporal relationship between cancer and SSc between 2 and 10 months. These patients all had invasive ductal breast carcinoma, with no distant metastases but nodal involvement in 4 of them. These patients all received surgical treatment, radiotherapy (*n* = 8), chemotherapy (*n* = 6) and hormonotherapy (*n* = 6). One patient exhibited ACA positivity, and one had ATA-scl70 positivity. Notably, it appeared that 7 on these 9 patients had antinuclear autoantibody with no defined specificity (dnATB) (neither anti-topoisomerase 1 nor anti-centromere antibody).

#### 3.3.2. Second Peak: Delayed Temporal Relationship between SSc and Cancer Diagnosis (10 Years ± 5 Years)

The second peak of cancer occurrence among SSc patients was around 10 years (±5 years) after SSc diagnosis. These twenty-two cancers were distributed as follow: 3 breast cancers, 4 lung cancers, 5 GI tract cancers (1 pancreas cancer, 1 ampullary cancer, 1 case of synchronous oesophageal and cholangiocarcinoma cancers, 1 colon cancer), 3 urinary tract cancers (1 kidney cancer, 1 urothelial cancer, 1 prostatic cancer), 2 ear, nose and throat (ENT) cancers, 2 lymphomas, and 3 skin cancers (basal cell carcinomas) (Figure 3). The main characteristics of SSc associated with these late-onset cancers are shown in Table 2, and the characteristics and outcomes of cancer events are shown in Figure 3. Cancer prognosis was poor for 8 of them (40.0%) due to 8 deaths (two patients lost to follow-up). None of them had a poor outcome due to SSc.

Five of them had received cyclophosphamide due to SSc before the onset of cancer (1 lung cancer, 1 urothelial cancer, 1 lymphoma, 1 ampullary cancer, 1 skin cancer, 1 squamous cell carcinoma of the palate). Four of them received azathioprine (1 breast cancer, 1 ampullary cancer, 1 lung cancer and 1 kidney cancer). Seven of them received methotrexate (2 breast cancers, 1 ampullary cancer, 1 kidney, 1 lymphoma, 1 colon and 1 prostatic cancer) (Supplemental Table S2b).

#### 3.3.3. Cancer Diagnoses Widely Spaced Apart from SSc Diagnosis

Cancers diagnosed before SSc (more than five years before). These concerned 11 cancers: 7 breast cancers, 2 skin cancers, 1 blood malignancy, and 1 endometrial carcinoma. Breast cancers included 7 patients (7 cancers) with a mean age at SSc diagnosis of 59 ± 8 years, and a mean age at cancer diagnosis of 43 ± 10 years. All these patients had complete remission of their breast cancer at the time of SSc diagnosis. Among these patients, 5 showed ACA positivity and 2 showed dnANA positivity. At the end of follow-up, one breast cancer status was unknown (lost to follow-up), and five patients were in complete remission for breast cancer (one patient died due to cancer of the pancreas 29 years after breast cancer).

Very delayed onset cancer after SSc (>15 years) concerned 3 breast cancers, 2 lung cancers, 2 skin cancers and one mesothelioma.

## 4. Discussion

### 4.1. Comparison of Cancer Distribution with Epidemiological Data from Literature

The proportion of cancers within our population is consistent with previous published series. Indeed, Shah and colleagues reported 168 patients with cancers among 1044 SSc patients (16.0%) [8], and Morrisroe and colleagues reported 245 cancers among a total population of 1721 SSc patients (14.2%) [9]. The observed higher median age at SSc diagnosis within the cancer subgroup was also reported by previous studies [8,9].

Our study of SSc patients revealed a majority of breast cancers (34.4%), followed by lung (14.1%), gastro-intestinal tract (12.5%) and skin (12.5%) cancers, spread over specific time periods. Breast and lung cancers are among the three most frequent cancers affecting women in France. Worldwide data from registries suggest a higher prevalence of cancer among SSc patients, and the proportions of each cancer in our study are similar to those of the literature. Our results are very close to those of Morrisroe and colleagues who found 245 cancers among 1727 SSc patients monitored from 2008 to 2015 including 30.2% with breast cancer, 11.8% with GI tract cancers, and 10.2% with lung cancer [9]. Data from our monocentric retrospective study share similarities with epidemiological data from literature, and also show certain specificities. A recent French retrospective study of 21 SSc patients found a similar proportion of breast cancers (28%), but a higher proportion of lung cancers (28%) [10].

In the present study, breast neoplasm was the most frequent type of cancer, particularly in the early years around the onset of scleroderma, and this is consistent with previous articles [11]. However, the overall increased risk of breast cancer incidence among SSc patients has not been demonstrated in all previous studies [12,13]. Although some studies suggest an over-risk of breast cancer within SSc patients [11,14,15], data from meta-analyses did not confirm this association [2]. These heterogeneous results may be explained by the heterogeneous management of patients (heterogeneity in the length of the follow-up of patients and in the determination of the antibody status). Notably, cancers diagnosed before SSc diagnosis are not always depicted. Moreover, in the studies focusing on long-term follow-up and late-onset cancers, the proportion of breast cancers may be reduced by a higher level of lung cancers and upper aero-digestive tract (UADT) cancers (tobacco and alcohol-related cancers) [2]. All these factors may contribute to the heterogeneity of data on breast cancers.

Our results concerning the proportion of lung cancer are similar to those previously reported. Hill and colleagues reported a higher prevalence of lung cancers among Australian SSc patients [16], like the data from Korean, Japanese, Taiwanese or Turkish patients [15,17,18,19]. Olesen and colleagues also reported a higher prevalence of tobacco and alcohol-related cancers (such as lung cancer) from a Danish cohort of SSc patients (2040 patients, 16,003 persons/year, 222 cancers) [12]. These data were confirmed by two meta-analyses demonstrating the increased relative risk (RR) of 4.35 (95% confidence interval (CI), 2.08–9.09), and standardized incidence ratio (SIR) of 3.18 (95%, CI 2.09–4.85) for lung cancers [2,13].

In the present study, we also observed other cancers associated with SSc, which had already been reported in literature at a lesser frequency: (i) UADT cancers [18,19,20]; (ii) skin cancers, whose prevalence among all cancers was higher in our study than in the study by Moinzadeh and colleagues (14.5% versus 3.8%), but similar to the Spanish study by Bernard-Bello and colleagues [3,21]; (iii) blood malignancies (non-Hodgkin’s lymphoma, myeloproliferative disorders, or myeloid leukemia [13,14,18]; (iv) and urothelial cancer [13]. However, we are unable to provide the prevalence of cancers among SSc patients in our series as we were focusing on SSc patients with cancer.

One of the strengths of our study was to document the temporal relationship between cancer occurrence and SSc onset. We observed two peaks of occurrence of cancer onset. The first peak is close to the diagnosis of SSc (±5 years), and the second takes place ten years later (±5 years). The first peak has already been described in literature, whereas the second one has not been described precisely.

### 4.2. First Peak: the Close Temporal Relationship between SSc and Cancer

The close temporal interplay between the onset of scleroderma and cancer (ranging from ±3 to ±5 years), has already been discussed in the recent literature and this relationship is more striking with regard to breast cancer [3,22]: Colaci and colleagues found a median time of 2.5 years (1–21)between the two conditions whereas Launay and colleagues found 11.5 months (0–288) [11,22]. This association could be the consequence of the screening for cancer in a patient newly diagnosed with SSc. This temporal interplay also suggests the paraneoplastic nature of SSc in certain cases associated with breast cancer.

More than the subtype of cancer, the immunological profiles of patients with concomitant cancer and SSc diagnoses is of interest. SSc patients with anti-RNA polymerase III (RNApolIII) autoantibody positivity exhibit a higher prevalence of cancer than patients with other subtypes of autoantibodies [3,8,23]. Moreover, SSc patients with anti-RNApolIII positivity had a greater risk of cancer close from SSs diagnosis (36 months to 5 years according to the studies) [3,5,24], especially concerning breast cancers [5,25]. Thus, acute screening of breast cancer is currently proposed among newly-diagnosed SSc patients with anti-RNApolIII antibodies [25].

In our retrospective study, we were unable to investigate the prevalence of anti-RNApolIII autoantibodies because this test was not routinely applied during the study period at our hospital. This obliged us to develop the concept of “double negative” patients (dnANA), i.e., without ACA or ATA-scl70 antibodies. This concept of dnANA may be interesting in clinical practice for two reasons: (i) all the current available laboratory kits do not include all the target antigens available today and are not themselves available in every centre among the world; (ii) each antibody specificity is mutually exclusive, as demonstrated by Patterson and colleagues in their large study [26]. This subgroup of patients exhibited a close temporal relationship between cancer events and SSc diagnosis. Apart from anti-RNApolIII, it is likely that certain other recently-discovered autoantibodies—biomarkers of early-onset cancer—are positive for this subgroup of dnANA patients. It is also likely that other autoantibodies remain to be discovered. Similarly, Igusa and colleagues reported that within SSc patients lacking ACA, ATA-scl70 or anti-RNApolIII autoantibodies (referred to as “CTP-negative” patients (CTP: Centromere, Topoisomerase, PolymeraseIII)), cancer risk was increased (SIR 1.83, 95% CI 1.10 to 2.86) [5]. A study from Xu and colleagues recently studied the sera of the same subgroup of SSc patients (“CTP-negative” patients), with a short time from cancer diagnosis to SSc onset (median 1.95 years). In four of them (25%), they identified a novel antibody targeting RNPC-3 (a protein of the minor spliceosome) [27].Thus, the concept of “dnANA” or “CTP-negative” patients, could be more appropriate to refer to a subgroup of patients with yet-undiscovered autoantibodies cross-reacting with tumor antigens and at risk of cancer within a short time of SSc diagnosis [27].

This intriguing time relationship between SSc and cancer and the particular subset of autoantibodies involved are two original features, stimulating research on the mechanisms between cancer and auto-immunity. Rosen and colleagues analyzed breast and ovarian tumors from SSc patients with anti-RNApolIII positivity, and observed a specific nucleolar expression of RNApolIII in malignant cells, compared to normal tissue and the tumors of SSc patients without anti-RNApolIII antibodies [28]. They then compared 8 tumors from ATA-scl70 antibody or ACA patients with 8 tumors obtained from patients with anti-RNApolIII antibodies (more precisely, antibodies directed against RPC1, a RNA-polIII subunit) [29]. Six out of eight tumors from anti-RPC1 positive SSc patients exhibited genetic alterations of the *POLR3A* gene (coding for RNA polymerase), while no mutation was found in the other patients’ tumors. So, these somatic mutations within tumor cells might initiate the autoimmune response and production of anti-RNApolIIII antibodies, targeting cancer cells. The authors also proved that anti-RPC1 autoantibodies from SSc patients not only targeted mutated antigens but also wild-type RNApolIII. Thus, autoantibodies initially directed towards the mutated antigen of the tumor may undergo an “epitope-spreading mechanism” leading to a cross-reactive recognition of a “normal” RNApolIII subunit, explaining the emergence of the immunological signature (RNApolIII antibodies) in the subgroup of SSc patients with cancer [30].

Interestingly, this over-reaction of the immune system against cancer may lead to a SSc phenotype and help to stop the spread of cancer. Yet, recently, Shah and colleagues described a particular subset of patients exhibiting RNA-PolIII antibodies, which target a specific subunit of RNApolIII (RPA194). These autoantibodies might have a “protective effect” against cancer as suggested by a higher prevalence among RNA-PolIII positive patients without cancer (16 positive sera in 88 SSc patients without cancer versus 3 in 80 patients with cancer) [31]. So, anti-RPA194 antibodies may stem from the development of cancer in a subgroup of patients [31], which is in line with the putative role of autoimmunity in the control of cancer spreading [32].

However, this immune strategy of “cancer-control” may not be sufficient to control the development of cancer related to high-speed mutating cells. Indeed, quick mutations can lead to a loss of expression of immunogenic antigens, and thus to an escape from the immune-system control [33]. Although it was not significant in our study, patients with a short delay between cancer and SSc diagnosis seem to have a more aggressive cancer (leading to death and/or metastatic stages) than patients whose cancer had been diagnosed long before the onset of SSc. This trend was already noticed by Launay and colleagues: in their review, 18 patients out of 33 (51.4%) died, including 11 patients (33.3%) in the first year after the diagnosis of breast cancer [22]. In these cases, we thus hypothesize that the immune system may have been overwhelmed by tumor spreading in the wrong way (autoimmunity rather that tumor destruction).

Whatever the reason, a short temporal relationship between SSc and cancer undoubtedly represents an interesting condition for exploring the complex interactions between the immune system and cancer development.

### 4.3. Second Peak: Late-Onset Cancers May Result from Multiple Intertwined Factors

The second peak of cancer occurrence among SSc patient concerns “late-onset” cancers. This concentration of cancer occurrences ten years after SSc diagnosis deserves to be considered. In our study, most of them are gastro-intestinal tract and lung cancers, but skin and urinary tract cancer occurrences are also represented. This distribution of cancers is consistent with the results from Onishi and colleagues [13]. Concerning lung cancers, this is consistent with the previous finding by Bonifazi and colleagues [2] who reported an average period of >5 years between SSc onset and lung cancer in their meta-analysis. Our findings are also consistent with the work from Shah and colleagues, who described a peak of cancers around 10 years after SSc diagnosis [8,34].

The emergence of late-onset cancers may be subtended by similar environmental and genetic background factors, which could lead to both cancer and SSc. Another explanatory condition is that preceding SSc may be a fertile ground for carcinogenesis. Therefore, several intertwined pathophysiological axes should be discussed, those involving endogenous and exogenous factors.

Among potential exogenous factors, chronic exposure to substances that are both carcinogenic and responsible for the development of scleroderma (such as crystalline silica and organic solvents) may be involved. Similarly, the use of certain SSc treatments may be involved in carcinogenesis: immunosuppressants used in SSc may explain an over risk of skin cancer, already well described among transplanted patients, especially concerning bladder or hematologic cancers [9,35]. In the present study, a significant number of “late-onset” cancers were associated with immunosuppressive treatments (including azathioprine and cyclophosphamide, Supplemental Table S2b). Calcium channel blockers (CCB) have also been proposed to explain an over-risk of cancer among SSc patients, especially breast cancer [11,21,36]. In their recent study of 1727 SSc patients, Morrisroe and colleagues found a significant difference in the use of calcium channel blockers between patients with cancer (*n* = 245) compared to patients without cancer (*n* = 1482), and especially in the case of prolonged use (*p* < 0.001, univariate analysis) [9].

Concerning endogenous factors, chronical inflammation, oxidative balance dysregulation, cytokine dysregulation or cell dysfunction are probably involved in cancer and SSc pathophysiology. Chronic inflammation experienced by some targeted organs among SSc patients is dramatically involved in cancer genesis [37,38]. Lung fibrosis and GERD—both hallmarks of SSc, with repeated tissue damage and repair, could both contribute to cancer development within these organs or others [9,39]. Moreover, local inflammation could also be involved in skin carcinogenesis in SSc, as suggested by Boozalis and colleagues, who observed a statistical association between scleroderma and skin cancer [40]. Oxidative stress, involved both in cancer [41] and the SSc pathophysiology [42], is probably maintained by this chronic inflammation. The occurrence of cancer 10 years after SSc diagnosis is compatible with a reasonable mean length of time for the effect of chronic inflammation or immunosuppressive drugs on carcinogenesis. Cytokine dysregulation may also play a role in both diseases: apart from angiogenesis factors, for example platelet-derived growth factor (PDGF) would appear to be involved in both carcinogenesis and lung fibrosis [43,44,45]. Lastly, abnormalities in mesenchymal cell homeostasis (fibroblast cells, endothelial cells), leading to both fibrosis and carcinogenesis, may also be involved in both carcinogenic and dysimmune processes [1].

Additionally, genetic and epigenetic alterations (such as telomere shortening, chromosomal instability, senescence, increased proliferation rates, immune deregulation and impaired cell metabolism), also contribute to the pathophysiology of both cancer and SSc [1].

Finally, the type of cancers described in this second peak are also compatible with the most frequent cancers within the same age and sex population.

All these factors, potentially involved in the pathophysiology of late-onset cancers, could also explain the high number of double cancers in our study (12 double cancers out of 55 patients, 21.8%). Our results are in line with those of Morrisroe and colleagues, who observed that 17.6% of SSc patients had more than one cancer [9]. Comparatively, among the 2,116,163 individuals from the general population of an American cohort study, only 8.1% patients had a second cancer [46]. The higher prevalence of double cancers in SSc might therefore also illustrate the contribution of the above-mentioned factors (i.e., endogenous and exogenous factors, and genetic or epigenetic factors).

### 4.4. Cancer-Screening Strategy: Current Recommendations and Additional Suggestions

As presented earlier, the first peak of cancer occurrences includes patients with “double-negative ANA” in the present study. As discussed before, data from literature report a significant proportion of patients with RNA-PolIII antibody positivity within this subgroup of “early-onset cancer”. Lazzaroni and colleagues proposed a specific cancer screening strategy within this subgroup of SSc patients with RNA-PolIII antibody positivity: breast cancer screening in female patients, and non-invasive tests or computed tomography (CT) scans guided by an extensive clinical evaluation in all patients [25]. Shah and colleagues proposed to perform an exhaustive cancer screening strategy for patients with newly diagnosed SSc presenting “red flags”, such as RNA-PolIII antibody positivity, especially in elder patients [34]. Our work suggests that the subgroup of patients that should benefit from an exhaustive cancer screening strategy would be the patients with newly diagnosed SSc, especially when presenting “double negative ANA”. This subgroup includes RNA-PolIII antibody positivity subgroup of patients, but also all the patients with autoantibodies targeting antigens still unavailable in commercial kits.

The originality of our work is that we highlight “late-onset cancers”, occurring about 10 years after SSc diagnosis. In the subgroup of SSc patients with prolonged inflammation targeting lung and gastro-intestinal tracts (such as SSc patients with GERD), especially after immunosuppressive treatments, particular caution should be exercised with regard to cancer onset. The confirmation of our results in a larger cohort with extended follow-up would be very important, since this condition appears crucial in clinical practice. In addition, because of the risk of second cancer, a particular attention should be paid to the subgroup of SSc patients who have already developed a cancer.

## 5. Conclusions

In the present study, we show two peaks of occurrences of cancers among our SSc patients. These peaks are associated with specific features and might be related to distinct mechanisms. As suggested by recent data from the literature, early-onset cancers (mainly breast cancers) might trigger the immune system, leading to an overimmune response and the onset of SSc. This subgroup is interesting in order to understand the immune system’s defence strategy against cancer and may offer directions for future research, including innovative therapeutic strategies. In clinical practice, this concept supports the need for tracking cancer in new-onset SSc (especially in those with “double-negative ANA”), as proposed previously [25,34]. Conversely, “late-onset cancers” (mainly gastro-intestinal tract and lung cancers) are probably favored by the subtle interplay between repeated target organ inflammation, immunosuppressants, mesenchymal cells homeostasis impairment and subsequent genetic alterations. The observation of these two peaks of cancer occurrences among SSc patients drives the physician to perform a specific cancer-screening strategy. In any event, many additional studies are required to document the association between cancer and SSc and to formalize cancer screening within the SSc population.

## Figures and Tables

**Table 1:** General characteristics of systemic sclerosis (SSc) patients with cancer.

SSc Patients with Cancer, *n*	55	
Median Age at SSc onset (IQR), years	56	(47–66)
Median Follow-up Time (IQR), years	11	(4–15)
Sex		
Female, *n* (%)	42	76.4
Male, *n* (%)	13	23.6
Tobacco Use, *n* (%)	19	34.5
SSc Subtypes		
lcSSc, *n* (%)	32	58.2
dcSSc, *n* (%)	20	36.4
Sine Scleroderma, *n* (%)	3	5.5
Organ Involvement		
Lung Fibrosis, *n* (%)	18	32.7
PAH, *n* (%)	8	14.5
SRC, *n* (%)	2	3.6
SSc-Cardiomyopathy, *n* (%)	3	5.5
GERD, *n* (%)	44	80.0
Digital Ulcer, *n* (%)	21	38.2
SSc Autoantibody Status		
ANA, *n* (%)	52	94.5
ACA, *n* (%)	16	29.1
ATA, *n* (%)	12	21.8
Double-Negative SSc Patients, *n* (%)	24	43.6
Use of Immunosuppressive Drugs Prior to Cancer		
Corticosteroid	19	34.5
Hydroxychloroquine	5	9.1
Methotrexate	12	21.8
Leflunomide	1	1.8
Mycophenolate Mofetil	2	3.6
Azathioprine	7	12.7
Cyclophosphamide	7	12.7
Biotherapy	1	1.8
Evolution		
Lost to Follow-up, *n* (%)	7	12.7
Death, *n* (%)	19	34.5
SSc-Related Death Cases	2	3.6
Malignancy-Related Death Cases	16	29.1
Other Cause of Death	1	1.8

SSc: systemic sclerosis; IQR: interquartile range; ANA: Antinuclear antibody; ACA: Anticentromere antibody; ATA: anti-topoisomerase 1 antibody; lcSSc: Limited cutaneous systemic sclerosis; dcSSc: Diffuse cutaneous systemic sclerosis; GERD: gastroesophageal reflux disease; PAH: pulmonary arterial hypertension; SRC: Scleroderma renal crisis.

**Table 2:** Characteristics of systemic sclerosis and overall outcome according to cancer subtype.

Characteristics	Cancer Subtypes														
	Breast (*n* = 22)		Lung (*n* = 9)		GI (*n* = 8)		Skin (*n* = 8)		Haemato. (*n* = 6)		Urinary (*n* = 5)		ENT (*n* = 3)	Gyn. (*n* = 2)	Meso. (*n* = 1)
**Sex**															
Female, %	22	100.0	2	22.2	6	75.0	7	87.5	4	66.7	2	40.0	2	2	1
Male, %	0	0	7	77.8	2	25.0	1	12.5	2	33.3	3	60.0	1	0	0
**Median Age at Cancer Diagnosis (years, IQR)**	55	IQR: 47–65	56	IQR: 56–65	60	IQR: 56–72	65	IQR: 49–74	68	IQR: 64–76	66	IQR: 54–67	55	71	76
**Median Age at SSc onset (years, IQR)**	59	IQR: 50–66	51	IQR: 43–56	58	IQR: 48–68	53	IQR: 49–61	63	IQR: 59–67	55	IQR: 54–62	47	69	39
**Tobacco Use, %**	5	27.8	6	75.0	3	50.0	2	25.0	1	20.0	4	80.0	2	0	NA
**SSc Subsets**															
lcSSc, %	14	63.6	3	33.3	5	62.5	4	50.0	6	100.0	4	80.0	2	0	0
dcSSc, %	6	27.3	6	66.7	3	37.5	4	50.0	0	0.0	1	20.0	1	1	1
Sine Scleroderma, %	2	9.1	0	0.0	0	0.0	0	0.0	0	0.0	0	0.0	0	1	0
**SSc Autoantibody Status**															
ANA, %	22	100.0	9	100.0	8	100.0	7	87.5	5	83.3	4	80.0	3	2	NA
ACA, %	10	45.5	0	0.0	4	50.0	3	37.5	1	16.7	2	40.0	0	0	NA
ATA, %	2	9.1	4	44.4	3	37.5	2	25.0	0	0.0	1	20.0	1	1	NA
dnANA, %	10	45.5	5	55.6	1	12.5	2	25.0	4	66.7	1	20.0	2	1	NA
**Metastatic Stage at Cancer Diagnosis** **(*n*, %)**	0	0	2	22	3	43	0	0	NC		0	0	1	0	0
**IS Treatment Prior to Cancer**	1/6		3/8		3/6		2/5		3/5		2/4		1/2		
**Overall Outcome**															
Death, %	5	22.7	5	55.6	7	87.5	3	37.5	2	33.3					
Metastasis, %	2	9.1													
Complete Remission, %	11	50.0	1	11.1	1	12.5	5	62.5	1	16.7	3	60.0	2	2	
Ongoing Treatment, %	3	13.6							2	33.3	2	40.0			
Lost to Follow-up, %	1	4.5	3	33.3					1	16.7			1		1

SSc: systemic sclerosis; lSSc: limited cutaneous systemic sclerosis; dSSc: Diffuse cutaneous systemic sclerosis; IQR: interquartile range; ANA: Antinuclear antibody; ACA: Anticentromere antibody; ATA: anti-topoisomerase 1 antibody; dnANA: double negative ANA; GI: gastro intestinal tract cancers; Haemato.: haematological cancers; ENT: ear, nose and throat cancers; Gyn.: gynaecological cancers; Meso.: mesothelioma; NA: data not available; NC: not concerned.

## Supplementary Materials

Supplemental tables (Table S1b and Table S2b) are available online at https://www.mdpi.com/2077-0383/9/3/853/s1. Table S1b: Patients with systemic sclerosis and two cancers. Table S2b: Immunosuppressive treatments received by SSc patients before cancer events.
