# Supplementary material for: Biphasic Temporal Relationship between Cancers and Systemic Sclerosis: A Clinical Series from Montpellier University Hospital and Review of the Literature
Source: J Clin Med. 2020 Mar 20;9(3):853. doi: 10.3390/jcm9030853 (PMC7141543; doi:10.3390/jcm9030853)
Supplement: Supplementary file 1 [file jcm-09-00853-s001.pdf]

Supplemental tables

Table 1b: Patients with systemic sclerosis and two cancers.

Patients	Age at SSc onset (years)	SSc subset	Cancer 1	Delay between SSc diagnosis (T0) and cancer #1 onset (months)	Cancer 2	Delay between SSc diagnosis (T0) and cancer #2 onset (months)
Patient 1	68	lcSSc	synchronous breast cancer	-252	synchronous breast cancer	0
Patient 2	58	lcSSc	breast	-240	pancreas	108
Patient 3	60	lcSSc	breast	-132	breast	84
Patient 4	64	lcSSc	breast	-6	myelodysplasia	38
Patient 5	45	lcSSc	melanoma	-190	breast	-3
Patient 6	66	dcSSc	colon	-27	lung	21
Patient 7	69	dcSSc	endometrium	-201	breast	40
Patient 8	62	lcSSc	B-cell lymphoma	12	prostate	48
Patient 9	73	lcSSc	basal cell carcinoma	95	ampulloma	111
Patient 10	47	dcSSc	oesophagus	120	cholangiocarcinoma	120
Patient 11	51	dcSSc	basal cell carcinoma	157	lung	165
Patient 12	18	lcSSc	synchronous lung cancer	312	synchronous lung cancer	0

lSSc, limited cutaneous systemic sclerosis; dSSc, diffuse cutaneous systemic sclerosis.

Table 2b: Immunosuppressive treatments received by SSc patients before cancer events.

	Breast cancers	Lung cancers	GI tract cancers	Skin cancers	Blood malignancies	Urinary tract cancers	ENT cancer	Total
Number of patients with cancers diagnosed after SSc	7	8	6	5	5	4	2	37
Patients with IS treatment prior to cancer event	2 (28.6%)	3 (37.5%)	3 (50.0%)	2 (40.0%)	3 (60.0%)	4 (100.0%)	1 (50.0%)	18 (48.6%)
MTX	2	2	3	2	2	2	0	13
AZA	1	3	1	1	1	2	0	9
CYC	0	2	1	1	2	2	1	9
LEF	0	0	0	1	0	0	0	1
MMF	0	0	0	0	1	0	1	2

SSc, systemic sclerosis; GI: gastro intestinal; ENT, ear-nose-throat; MTX, methotrexate; AZA, azathioprine; CYC, cyclophosphamide; LEF, leflunomide; MMF, mycophenolate mofetil; IS, immunosuppressants.
